# The Suppression of Irrelevant Semantic Representations in Parkinson’s Disease

**DOI:** 10.3389/fnhum.2018.00511

**Published:** 2019-01-22

**Authors:** Megan L. Isaacs, Katie L. McMahon, Anthony J. Angwin, David A. Copland

**Affiliations:** ^1^Centre for Clinical Research, University of Queensland, Herston, QLD, Australia; ^2^School of Health and Rehabilitation Sciences, University of Queensland, Saint Lucia, QLD, Australia; ^3^School of Clinical Sciences and Institute of Health and Biomedical Innovation, Queensland University of Technology, Brisbane, QLD, Australia

**Keywords:** negative-priming, inhibition, Parkinson’s disease, lexical-semantics, language

## Abstract

The impairment of lexical-semantic inhibition mechanisms in Parkinson’s disease (PD) remains a source of contention. In order to observe whether people with PD are able to suppress irrelevant semantic information during picture naming, the present study employed an object-based negative priming paradigm with 16 participants with PD and 13 healthy controls. The task required participants to name a red target image while ignoring a superimposed, green distractor image. The semantic relationship between the distractor image and the target image of the subsequent trial was manipulated, such that the distractor image was identical, semantically related, or semantically unrelated to said target image. The PD group and the control group were slower in naming a target image that had previously served as a distractor image, relative to naming a target image that was unrelated to the previous distractor image. Thus, a negative priming effect was present in both groups. Furthermore, no significant difference in the magnitude of this effect was observed between the control and PD groups. When considered in the context of existing literature surrounding negative priming in PD, these results suggest that inhibition is subserved by multiple, domain-specific mechanisms and that the inhibitory processing of visual-semantic stimuli is intact in PD.

## Introduction

Parkinson’s disease (PD) may influence the inhibition of inappropriate or irrelevant stimuli, however, this issue is a point of contention. Even within the body of work that suggests impairment *is* present, the magnitude and nature of the disruption varies considerably between paradigms and modalities (Gauggel et al., 2004; Bokura et al., 2005; Grande et al., 2006; Seiss and Praamstra, 2006; Obeso et al., 2011). Shao et al. (2015) note that inhibition is a general term used to refer to a large number of processes recruited under specific circumstances. The particular inhibitory processes affected in PD remain a subject of debate, and tasks that can reliably isolate different aspects of inhibition are required for further illumination.

The lack of agreement concerning inhibitory processing in PD is readily manifest in the literature concerning lexical-semantic mechanisms, which is the focus of the present study. Many studies have provided evidence for altered performance across a variety of lexical-semantic tasks including verbal fluency (Auriacombe et al., 1993; Tröster et al., 1998; Piatt et al., 1999; Henry and Crawford, 2004; Herrera et al., 2012), semantic priming (Murdoch et al., 2000; Arnott et al., 2001; Copland, 2003; Filoteo et al., 2003; Angwin et al., 2009), and confrontation naming (Cotelli et al., 2007; Rodríguez-Ferreiro et al., 2009). Disrupted semantic inhibition may represent a common underlying deficit that can account for these impairments.

It may be posited that these issues in lexical-semantic inhibition reflect a broader deficit in ignoring irrelevant stimuli, and attempts have been made to develop paradigms that test this hypothesis. Negative priming tasks provide a useful tool for examining the influence of ignored distractors over time. Tipper (1985) was the first to provide an account of the classic object-based negative priming paradigm. In this task, participants were presented with two line drawings superimposed over each other, one colored red and the other green. They were asked to name the red image and ignore the green image. The relationship between the green distractor image and the red target image of the subsequent trial was manipulated such that the green distractor was either unrelated, semantically related, or identical to the subsequent red target. Tipper found that when administered in healthy controls aged 18–45 years, this task elicited a negative priming effect. That is, when the distractor item presented with the prime was either identical or semantically related to the probe, the naming of the probe was slowed. This effect is assumed to occur as a result of inhibition processes called into play to suppress the representation of the distractor, thus allowing for naming of the prime that shares the display with the distractor. When the subsequent probe is identical to this distractor, the residual inhibition must be overcome in order to retrieve the appropriate response, thereby slowing responses to the probe word. This spreading inhibition account of negative priming has its root in theories of spreading lexical activation (Collins and Loftus, 1975; Roelofs, 1992; Levelt et al., 1999). Of note, a number of semantic priming studies in PD cohorts have provided evidence for reduced competitive inhibition in this population (Gurd and Oliveira, 1996; Copland, 2003; Arnott et al., 2010). That is, these individuals have difficulty inhibiting unwanted information—a finding that would appear to suggest that this population may encounter difficulty in a negative priming task.

When considering the processes central to execution of negative priming tasks, it is also relevant to acknowledge the influence of executive functions such as set-switching. Responding to an item that was previously ignored requires a shift in set between the rule “ignore” to the rule “attend.” Given that individuals with PD have consistently demonstrated impairment on tasks measuring set-shifting ability (Cools et al., 2001; Woodward et al., 2002; Monchi et al., 2004; Bokura et al., 2005; Lange et al., 2016; for systematic review see Kudlicka et al., 2011), interpretation of their performance on a negative priming paradigm must consider this potential confound.

Previous studies of negative priming in the PD population have generally manipulated visuospatial stimuli, observing the processing of location (spatial) and identity (object) features. In these paradigms, participants are generally required to respond to a pre-determined target stimulus using a manual point/touch response (e.g., identify the peripheral shape that matches the central shape, identify the central figure, or identify the “0”) whilst ignoring a distractor. Stimuli may be numbers or letters (e.g., Troche et al., 2006), or shapes (e.g., Wylie and Stout, 2002). In the subsequent trial, the target may share identity, spatial location, or both of these features with the previous distractor stimulus. When administered in PD cohorts, results have rarely been replicated across studies. Stout et al. (2001) found evidence for enhanced negative priming in PD on a visuospatial task, and Wylie and Stout (2002) later replicated these findings. The latter study reported enhanced negative priming in PD relative to controls for location, identity and location-identity conditions, suggesting that people with PD have greater difficulty overcoming residual inhibition compared to controls. In contrast, Filoteo et al. (2002) administered a visuospatial negative priming task to PD and healthy control groups, and found no evidence of any negative priming effect in the PD participants, despite its presence in the control group. Likewise, Troche et al. (2006) found no evidence of negative priming in control or PD groups when identity was manipulated, however both groups recorded a significant negative priming effect for trials where location was manipulated. This inconsistency across studies has sparked some commentary and the suggestion that this discrepancy could be explained by differences in a number of design features, including the nature of the stimuli and the response demands of the task (Stout et al., 2002).

Possin et al. (2006) further evaluated the lack of agreement in the literature around attention/inhibition tasks in PD and noted that much of the contention appeared to surround the distinction between spatial processing and object or identity processing. Possin et al. (2006) proposed that attention/inhibition is not a unitary mechanism, and that specialized components exist that can be impaired or spared independently. These authors developed a task to assess object-based attention in isolation from location-based attention. Their task used picture-based stimuli depicting common objects, similar to Tipper et al.’s (1985) procedure, and participants were shown a target and comparator and asked to indicate whether these two objects were the same or different. A distractor image was presented in the same display, slightly overlapping with the target. The target image was denoted by color. PD participants performed at a level commensurate with controls across all conditions, demonstrating an equivalent degree of negative priming in the ignored repetition (analogous to the present study’s identical condition). The authors interpreted this to suggest that object-based attention processes are intact in PD, while spatial (location) processing may be disrupted.

Marí-Beffa et al. (2005) also employed a negative priming task to observe the processing of irrelevant stimuli in people with PD and healthy age-matched controls, however their paradigm utilized orthographic, rather than picture-based stimuli. They created a lexical decision task that manipulated the semantic relatedness between a distractor word in the prime display, and the subsequent target word in the probe display. Participants were encouraged to ignore the peripheral distractor word, and the probe display was presented once the participant had responded to the prime such that the stimulus-onset asynchrony (SOA) was variable across trials. Unrelated prime distractor and probe pairs were compared with either semantically related pairs (Experiment 1) or identical pairs (Experiment 2). In the first experiment, trials where the prime distractor was semantically related to the probe were significantly faster compared to unrelated trials in the PD group, however no significant priming was evident in controls. In the second experiment, the PD group showed significant positive priming for trials where the distractor was identical to the subsequent probe, while the control group showed significant negative priming under this condition. The authors suggested that these findings support the proposal that the underlying cause of the facilitatory priming effect observed in the PD group was a failure to successfully ignore the irrelevant distractor items, thus allowing these representations or their semantic relations to be more rapidly retrieved in subsequent probe trials.

Appraisal of the negative priming literature identifies several elements of task design that may account for the discrepancies in findings when administered to PD cohorts. Variation in characteristics such as input modality (e.g., visuospatial/location vs. picture stimuli vs. written word) and response requirements (non-verbal manual response vs. lexical decision vs. overt naming) emerge as potential points-of-difference across studies that report contrasting findings. The present study therefore administered a classic negative priming task (Tipper, 1985) that used a picture-in-picture paradigm to determine whether people with PD are able to inhibit irrelevant semantic information. The task required participants to respond to target images in the presence of distractors that were identical, related, or unrelated to the subsequent target. The manipulation of semantic relatedness was intended to allow for observation of the level to which distractor items are processed. Unlike the paradigms employed by Marí-Beffa et al. (2005) and Possin et al. (2006), the present task asked participants to name images aloud, necessitating recognition of visual object features, and subsequent access to the semantic and lexical information. This alteration may better inform conclusions regarding inhibition process as they specifically relate to lexical-semantics. The target image was superimposed over the distractor image and differentiated by color. Participants were required to respond to each trial in order to ensure blinding to prime or probe status. Each superimposed target-distractor image was displayed briefly before being replaced by a mask, with the intention of minimizing controlled processing of the image. It was hypothesized that the PD participants would show faster reaction times for targets that were semantically related or identical to the preceding distractor item as a result of difficulty suppressing irrelevant semantic information [in line with the findings of Marí-Beffa et al. (2005) and existing evidence from semantic priming studies in this population e.g., Copland (2003) and Arnott et al. (2010)], while the control group would demonstrate a negative priming effect (slower response times for related and identical targets). While the identical condition will examine suppression of the visual-semantic representation and its lexical form, slowing of related targets will be consistent with inhibition within the lexical-semantic network.

## Materials and Methods

The study was carried out in accordance with the recommendations of the 2007 National Statement on Ethical Conduct in Human Research, National Health and Medical Research Council (NHMRC). The protocol was approved by the University of Queensland Human Research Ethics Committee. All subjects gave written informed consent in accordance with the Declaration of Helsinki. Financial reimbursement was provided to all participants.

### Participants

Sixteen adults [nine females, mean age = 62.9 (6.3) years, mean years of education (YOE) = 13.6 (3.7)] with a diagnosis of idiopathic PD (diagnosis confirmed by a neurologist using Calne et al’s., 1992 criteria) were recruited from the community. Participants were right-handed, confirmed with the Annett Hand Preference Questionnaire (Annett, 1970), with English as a first language and no history of neurological surgery, trauma or substance abuse, or severe dysarthria impacting intelligibility. Fifteen neurologically healthy adults were initially recruited to act as controls, however during subsequent data analysis it was noted that two participants from this group presented with 10 or fewer valid trials (where a response was provided) across multiple conditions and were excluded from further analysis. A total of 13 control participants were therefore included in the final statistical analysis reported below [seven females, mean age = 65.9 (8.9) years, mean years of education (YOE) = 15.9 (2.9)]. This group was matched to the PD group for age (*p* = 0.3), gender (*p* = 0.9), and years of education (*p* = 0.81). Control participants were also right-handed (Annett, 1970), with no history of neurological disease, surgery, trauma, or substance abuse. All participants had normal or corrected-to-normal vision and hearing.

Participants in the PD group completed the PD Cognitive Rating Scale [PD-CRS; Pagonabarraga et al., 2008; mean total score = 105.5 (10.5)]. Those who achieved a score below 64 were excluded from further involvement in the study, as this score is considered to be indicative of significant cognitive impairment or dementia (Kulisevsky and Pagonabarraga, 2009). The Montreal Cognitive Assessment (MoCA v7.1; Nasreddine et al., 2005) was employed as a basic cognitive screener in order to broadly detect the presence of cognitive impairment in the control group [mean total score = 27.5 (2.8)]. Control participants were required to score within the normal range (±1 SD) for their age as identified by Rossetti et al. (2011) in order to be included in the study. The use of the norms generated by Rossetti et al. (2011) were favored over the cut off value of 24 originally identified by the authors of the MoCA (Nasreddine et al., 2005), as the former were derived from a large, ethnically-diverse population and accounted for age and level of education. The Geriatric depression scale (GDS; Sheikh and Yesavage, 1986) was administered to all PD participants [mean score = 2.4 (3.1)]. A score greater than eight on the GDS is considered indicative of major depressive disorder in PD (Dissanayaka et al., 2007, 2011), hence those participants scoring in this range were excluded unless they reported current use of anti-depressant medication or other medical treatment. A total of five participants with PD were taking anti-depressant medication at the time of testing. The PD participants had a mean Hoehn and Yahr rating (Hoehn and Yahr, 2001) of 2.1 (0.3). Levodopa Equivalent Daily Dosage (LEDD) was calculated for each PD participant according to the methods outlined by Tomlinson et al. (2010) [mean LEDD mg/day = 520 (383.44)].

### Experimental Design and Stimuli

A picture in picture task was designed to elicit semantic inhibition as a result of simultaneous presentation of a red line drawing superimposed over a green line drawing. Participants were required to name the red image aloud as quickly as possible and ignore the green image. A superimposed pair of images collectively referred to as the prime was presented first (red prime image and green distractor image), followed by the corresponding probe pair (red probe image and green distractor image). Stimuli were drawn from the International Picture Naming Project (IPNP) database (Szekely et al., 2004). The converged red and green images were created using Adobe CC Photoshop software (v2014.4.0), with the red image superimposed over the green image. The task design manipulated the relationship between the red target item in the probe pair and the green distractor item in the prime pair immediately preceding it.

**Figure 1:**
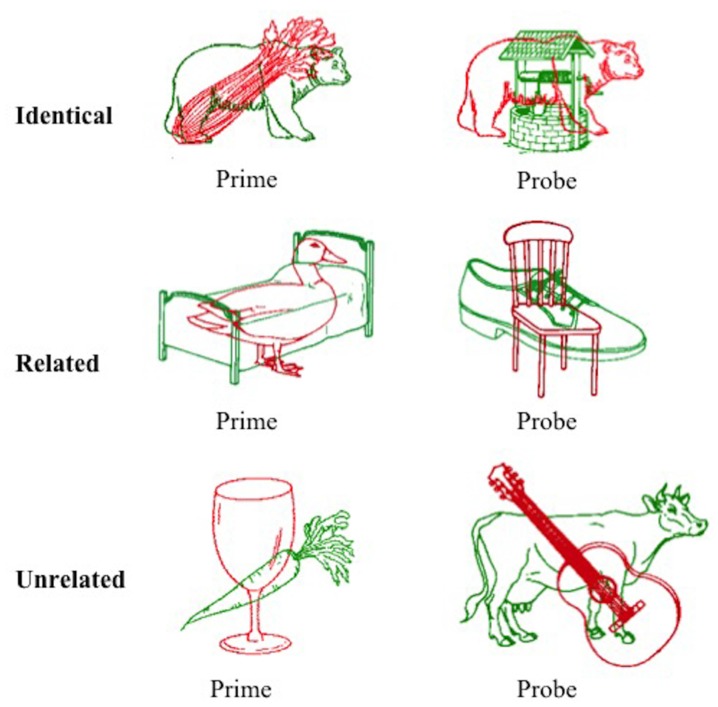
Example of prime and probe stimuli from the three conditions of the negative priming task. Participants were required to name the red item and ignore the green item. Images were adapted from the International Picture Naming Project, see Szekely et al. (2004).

A total of 144 superimposed images were developed, each containing a red target image and a green distractor image. For the purposes of this text, one superimposed image is referred to as one trial. These superimposed images were divided equally into three condition sets (identical, related, and unrelated), each containing 48 trials (with 24 pairs of primes and corresponding probes). The green distractor image in the prime stimulus was identical to the red image in the subsequent probe stimulus in the identical condition, semantically related to the red probe image in the related condition (achieved by selecting images from the same semantic category), or semantically unrelated in the unrelated condition (achieved by ensuring the two images were from distinct semantic categories as judged independently by two researchers). An example of stimuli from each condition is provided in Figure 1. The mean naming latency (IPNP; Szekely et al., 2004) and the Center for Lexical Information (CELEX) spoken word frequency (obtained from the N-Watch Database; Davis, 2005) of stimuli in each condition are presented in Table 1. These values did not differ significantly between the conditions (*p* = 0.173 and *p* = 0.592, respectively), though it should be acknowledged that there was a large degree of variability in CELEX spoken word frequency between conditions.

**Table 1:** Psycholinguistic properties of picture stimuli.

Condition		CELEX spoken word frequency	Naming latency (ms)
Identical	*M*	82.46	1,010
	*SE*	70.98	25
Related	*M*	16.96	952
	*SE*	4.36	19
Unrelated	*M*	12.28	952
	*SE*	5.72	23

*Naming latency obtained from the International Picture Naming Project, see Szekely et al. (2004). CELEX (Centre for Lexical Information) spoken word frequency obtained from the N-Watch database (Davis, 2005)*.

### Procedure

Stimuli were presented using a laptop with the screen set to 640 × 480 bit-depth resolution and positioned approximately 60 cm from the seated participant. The experiment was realized using Cogent graphics software (Wellcome Department of Imaging Neuroscience, 2013) *via* a Matlab platform (MathWorks, 2013). The task involved the presentation of superimposed red and green line drawings on a white square of 300 × 300 pixels in the center of the screen. A trial started with a fixation cross that was displayed for 100 ms followed presentation of the prime (superimposed red and green image) for 500 ms. A mask was then displayed immediately following presentation of this prime for 5,000 ms in order to discourage controlled processing of the images. This mask was a nonsensical image made from multiple line drawings superimposed over each other in red and green, such that no individual shape or picture could be easily discerned. During this 5,000 ms period, the subject was required to name the red image depicted in the prime as quickly as possible and the audio was recorded using a headset microphone. The task was self-paced, requiring participants to press the space bar after providing their response in order to progress. The task would move on automatically if the space bar was not pressed after the 5,000 ms response window had lapsed. The SOA was therefore variable across trials. Following either the provision of a response or the lapse of the 5,000 ms response window, a fixation cross was again displayed for 100 ms followed by presentation of the corresponding probe (superimposed red and green image). After 500 ms this probe image was replaced by the mask (described above). Participants were again required to verbally name the red image during the 5,000 ms period in which the mask was displayed, and the task was moved on *via* space bar press or the lapse of 5,000 ms. This pattern of prime-mask-probe-mask presentation was maintained throughout the experiment.

Three alternative pseudo-randomizations of stimuli were generated to minimize order effects. The randomization ensured that a prime-probe sequence from a given condition did not follow a sequence from the same condition (e.g., a prime-probe sequence from the identical condition could only be immediately followed by a related or unrelated prime-probe sequence). The experiment was conducted in a quiet room with minimal environmental distractions. The task was completed in one run with no rest breaks between trials and took approximately 20 min.

## Results

### Scoring

Response times were manually extracted from the voice recordings and were measured from the onset of the picture stimulus to the onset of the participant’s response. Accuracy was scored by two independent raters according to the following criteria: a correct response required the red image to be accurately named in a single word utterance (items with a two-word name e.g., washing machine were also permitted). Any response that contained multiple words, excessive interjections or false starts, self-corrections, or inaccurate names was scored as incorrect. Cohen’s kappa (κ) was run to determine the inter-rater agreement and results indicated an acceptable level of agreement, *κ* = 0.956 (95% CI 0.95, 0.96), *p* < 0.001.

### Behavioral Results

Of the total trials, non-responses (where no attempt to name the item was made) accounted for 18.2% in the PD group and 12.9% in the Control group. Of the remaining trials, only those in which naming latency was between 250 ms and 2,500 ms were included in the latency and accuracy analysis. These limits were included in order to avoid anticipatory errors and minimize the influence of controlled processing, similar to the extreme outlier method utilized by Marí-Beffa et al. (2005). As a result, 1.7% of these trials in the PD group and 0.98% of these trials in the control group were discarded.

#### Naming Response Time

Only those trials for which both the prime and the probe met criteria for a correct response were included. This resulted in discarding 24.7% of trials in the PD group and 16.9% of trials in the control group. An independent samples *t*-test confirmed that the difference in error rates between groups was not significant [*t*_(27)_ = 1.757, *p* = 0.09). A Shapiro-Wilks test of normality demonstrated that the data for both PD and control groups was not normally distributed (*p* < 0.001) and visual inspection revealed extreme positive skewness. A reciprocal transformation (1/x) was performed. Skewness and kurtosis figures indicated that this transformation substantially improved the distribution of the data for each group (PD Skewness z-score = 0.07, Kurtosis z-score = −1.5; Control—Skewness z-score = 0.46, Kurtosis z-score = −0.28). Transformed response times for probe trials were submitted to a Linear Mixed Model (LMM). Fixed effects were group (PD and control) and condition (related, unrelated, and identical). Participant was included as a random effect.

**Figure 2:**
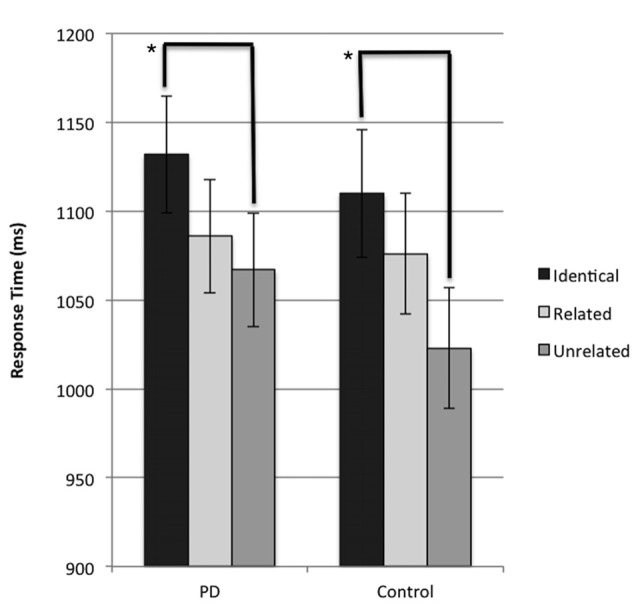
Mean response time (ms) for probe responses in identical, related, and unrelated conditions for each group. Brackets indicate significant differences (*p < 0.05). Error bars indicate mean standard error.

The results of the LMM revealed a significant main effect of condition for response time (*F*_(2,1340)_ = 7.351, *p* = 0.001). A Bonferroni-corrected pairwise comparison was performed and identified that when response time was collapsed across groups, participants were significantly faster (*p* < 0.001) in naming the target image in trials where the preceding prime distractor image was unrelated to the subsequent target probe image (unrelated condition), compared to trials where the preceding prime distractor was identical to the subsequent target image (identical condition). This result suggests the presence of a negative priming effect across both groups. Further analysis confirmed that this significant difference in response time between unrelated and identical conditions was present independently in both the PD and control groups (*p* = 0.02 and *p* = 0.027 respectively). These results are presented in Figure 2 in their untransformed state, for ease of interpretation. No significant difference between the related and unrelated condition was observed in either group (*p* > 0.05 for all comparisons). No main effect of group (*p* = 0.782) or significant interaction (*p* = 0.811) was observed.

##### Prime Trials

In order to confirm the validity of the negative priming effect detected for both groups in the analysis of response times for probe trials, the transformed response times for correct prime trials were also submitted to an LMM with group modeled as a fixed effect and participant number as a random effect. Results of this analysis demonstrated no group differences in response time for prime trials (*p* = 0.729).

##### Naming Accuracy

**Figure 3:**
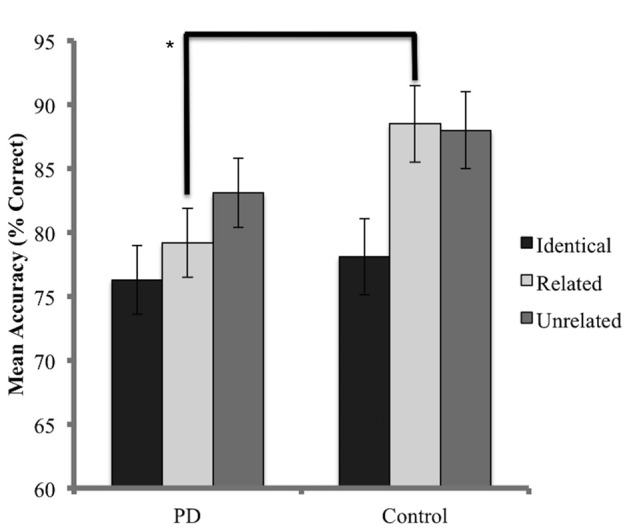
Mean accuracy (percentage correct) for probe responses in identical, related, and unrelated conditions for each group. Brackets indicate a significant difference (*p < 0.05). Error bars indicate mean standard error.

The accuracy analysis considered probe trials for which the prime was named correctly. As a result, 24.4% of trials for the PD group and 16.6% of trials for the control group were discarded. A Shapiro-Wilks test indicated that the data for the PD group was normally distributed (*p* = 0.41) while the control group did not achieve normality (*p* = 0.001) and data transformation was not successful in resolving this issue. Skewness values for the control group were also considered to be beyond an acceptable range (Skewness z-score = −3.4, Kurtosis z-score = 2.3). Non-parametric methods were therefore employed. Kruskal-Wallis tests, conducted independently for the PD and control groups, identified no significant difference in accuracy (percentage of target trials named correctly) between conditions (PD *x*^2^ = 4.68, *p* = 0.09; control *x*^2^ = 1.82 *p* = 0.4). When each condition was analyzed independently, Mann-Whitney *U* tests identified no significant differences between groups for accuracy in both the unrelated (*U* = 72, *p* = 0.16) and identical conditions (*U* = 87, *p* = 0.48). A significant difference in accuracy was detected in the related condition, favoring the control group (*U* = 51, *p* = 0.02). Results are presented in Figure 3.

## Discussion

The present study used an object-based negative priming task to determine whether people with PD are able to inhibit irrelevant semantic information. The PD group performed similarly to controls across all conditions in terms of naming latency, demonstrating that the retrieval of an object’s name was slowed when that same item had previously been ignored. This finding suggests that the ability to suppress irrelevant semantic information was intact in PD participants. A negative priming effect was not detected in either group when the target image was semantically related to the preceding distractor.

The results obtained for the control group are in line with existing literature concerning negative priming of objects, where response time is consistently slower for trials where the distractor in the prime display is identical to the subsequent probe (Tipper, 1985; de Zubicaray et al., 2006; Schrobsdorff et al., 2012). Furthermore, it has been recently established that identity or object-based negative priming is not influenced by age and the effect appears to remain constant across the lifespan (for reviews see Gamboz et al., 2002; Frings et al., 2015). It is therefore appropriate to make such comparisons between the results found in the present study, and studies in younger healthy populations.

The finding of consistent performance between PD and control groups with regard to the processing of ignored images is consistent with that of Possin et al. (2006), described above, who also manipulated stimuli presumed to activate visual-semantic representations. However, contrary to the present hypothesis, such a performance is in opposition to the results described by Marí-Beffa et al. (2005) in their lexical-decision based negative priming task, and to those obtained when the visuospatial version of the task is administered in this population (Stout et al., 2001; Filoteo et al., 2002; Wylie and Stout, 2002; Troche et al., 2006). Furthermore, a lack of impairment in lexical-semantic inhibition is in contrast to evidence provided by semantic priming studies, described above, suggesting that competitive inhibition is reduced in individuals with PD (e.g., Copland, 2003; Arnott et al., 2010). However, Possin et al.’s (2006) suggestion that attention/inhibition processes are not represented by a single mechanism goes some way toward explaining these conflicting findings. As previously discussed, Possin et al. (2006) concluded that object-based attention processes are intact in PD, while spatial (location) processing may be disrupted. After evaluating the vast catalog of negative priming studies since the emergence of the negative priming paradigm in 1966, a review by Frings et al. (2015) reached a similar conclusion. These authors proposed that it is inappropriate to make comparison between studies of negative priming in spatial-location and identity/object-based paradigms, as it appears likely that the mechanisms underlying each may differ to some degree. Indeed, the possibility of multiple, independent attention/inhibition mechanisms is a notion supported by a growing cohort of publications (Miyake et al., 2000; Nigg, 2000; Grande et al., 2006; Shao et al., 2015). If each type of inhibition is affected differentially in PD, this could explain the inconsistent performance observed across paradigms. The results of the present study certainly appear to support this conclusion, however further investigation is required regarding this hypothesis. It may therefore be of value to consider the present results within the domain of cognitive-linguistic processing and hence, lexical-semantic inhibition. This view may allow for speculation as to an alternative explanation for the differences found between the performance of people with PD on the present picture-based negative priming task, and Marí-Beffa et al.’s (2005) word-based negative priming task.

PD participants were observed to perform at a level commensurate with controls in the present study in terms of latency and negative priming, while PD participants in Marí-Beffa et al.’s (2005) study demonstrated positive priming under circumstances where controls demonstrated either no priming at all, or negative priming. Two key factors that warrant consideration when examining the differences between the present study and that of Marí-Beffa et al. (2005) are the use of picture-based stimuli, and the response requirement. First, the current study required naming of a target picture, in the presence of a distractor image. In contrast, Marí-Beffa et al.’s (2005) task only used word-based stimuli for both targets and distractors, and required a yes/no button press regarding the lexicality of the prime or probe target. As previously discussed, Stout et al. (2002) commented on the importance of considering differing response input and output modalities in the visuospatial domain. It may therefore also be appropriate to consider the possibility of a similar effect in the lexical-semantic domain. Furthermore, the reviews of negative priming conducted by Fox (1995) and later by Frings et al. (2015) concluded that “…ignored items are analyzed to the level of representation that is required by the task.” (Fox, 1995, p. 9). Frings et al. (2015) went on to suggest both retrieval and inhibition processes play a role in successful completion of negative priming, but that each may be recruited to different degrees depending on the task design. Balota et al. (2001) have also suggested that differing task goals will engage distinct processing pathways and that these can impact performance downstream. It is therefore important that task design is considered when interpreting the present results.

Indeed, the differing input modalities and response requirements in the Marí-Beffa et al. (2005) task and the present task may have given rise to the contrasting results. For example, in Marí-Beffa et al.’s (2005) task participants were required to ignore written distractors in order to make a lexical decision about a central target word. It has been demonstrated that participants with PD have difficulty inhibiting automatic word reading processes (Henik et al., 1993). On this basis, it could be assumed that the PD group were unable to effectively ignore the written distractor words in the task, and that these lexical units activated their semantic representations. It has been suggested that PD participants also have reduced lateral competitive inhibition (Gurd and Oliveira, 1996; Copland, 2003; Arnott et al., 2010). It is therefore possible that the activation of distractor word representations also spread to related concepts. However, it may be argued that completion of the lexical decision task does not necessitate the suppression of the distractor representation, as this may not interfere significantly with the lexical level of processing. PD participants are then able to make a successful lexical decision, but the additional activation of the distractor and its related concepts allows for speeded decisions when these words are repeated as the target in the subsequent probe display. In contrast, controls are able to effectively ignore the distractor words, or at least do not automatically process them to a categorical (semantic) level as the task requirements do not necessitate accessing the semantic system. They therefore demonstrate no significant priming effect for related trials, and demonstrate negative priming for repeated trials (where the distractor becomes the target) due to previous inhibition of the word form at a lexical level, not its semantic representation.

Comparatively, the use of a picture-naming design in the present study evokes different processing pathways. Visual stimuli access the semantic system following perceptual feature analysis (Humphreys and Forde, 2001), and this must take place prior to retrieval of lexical representations (also see Morton, 1980; Lesser and Milroy, 1993; Kay et al., 1996). Several authors have also demonstrated that abstract representations are still accessed for ignored objects (Dell’Acqua and Grainger, 1999; Morgan and Meyer, 2005) and indeed this has been demonstrated in object/identity based negative priming paradigms (Tipper and Driver, 1988; de Zubicaray et al., 2006). It may therefore be suggested that in the present study, both control and PD groups automatically access the abstract representation of the *ignored* object in the semantic system. However, the semantic representation of the *target* object must also be accessed in order for its name to be retrieved. This task requires the representation of the distractor to be inhibited, in order to resolve competition at the semantic level and allow the target image to be named. The present study demonstrated that the PD group were capable of executing this deliberate suppression. Both groups then experienced delayed naming latency and increased errors when they had to subsequently name this distractor in the probe display.

The notion that deficits in negative priming in PD relate to the demands of the task design, and not to a specific impairment in semantic inhibition, gains some support from the semantic priming literature. Specifically, a large body of semantic priming literature that frequently reports disruptions to inhibition processes in PD and such research typically employs lexical decision tasks (McDonald et al., 1996; Arnott et al., 2001; Copland, 2003; Angwin et al., 2005; Castner et al., 2007; Boulenger et al., 2008; Fernandino et al., 2013). Examining multiple tasks with contrasting demands within the same PD cohort would further verify this account.

An alternative explanation for the present findings may relate to the use of color-cues in the present study. It has been demonstrated in the motor realm that people with PD appear to benefit from external cues and demonstrate better performance on externally cued tasks relevant to internally cued tasks (Lim et al., 2005; Spaulding et al., 2013; Rocha et al., 2014). Brown and Marsden (1988) employed a variation of the Stroop task to demonstrate that PD participants were more impaired when the task demanded greater internal control. These authors suggested that impairment of cognitive functions like inhibition emerge when task demands exceed the capacity of the supervisory attentional system, and that the resources of the system were reduced in PD. In addition to using images as stimuli, the present study also provided a color-cue. Participants were always required to name the red image and ignore the green. In contrast, the lexical decision task used by Marí-Beffa et al. (2005) required internal evaluation and generation of a yes/no response. These differing task requirements may therefore also have contributed to the results observed.

It must be acknowledged that the semantic nature of the negative priming effect, as it is described here, remains somewhat contended in the literature. In an fMRI study of negative priming that used a similar paradigm to the current study, de Zubicaray et al. (2006) observed increased left anterior temporal cortex activity in the repetition-ignored condition (analogous to the present study’s identical condition). This region of the brain is generally thought to be responsible for the processing of abstract semantic representations (e.g., see Price et al., 2005), hence de Zubicaray et al. (2006) proposed that its activation demonstrated that ignored stimuli are automatically processed to this level. This may be interpreted as evidence for the semantic nature of the negative priming effect, however, alternative explanations have also been proffered, including a possible locus in memory encoding mechanisms (Neill et al., 1992; Milliken et al., 1998; Mayr and Buchner, 2007). Certainly, the present study did not observe evidence of delayed processing of ignored stimuli that were semantically related to the target image in either the PD or the control group, which may reflect limited processing of distractors beyond the automatic activation of visual-semantic representations. However, Damian (2000) have suggested that elicitation of a negative priming effect for related items may relate to the degree of semantic association between the distractor and the target (a factor that was not controlled in the present study). Future investigations would benefit from the systematic manipulation of semantic relatedness between distractor and target, in addition to the utilization of fMRI to observe whether any resulting naming delay is associated with activation in neural regions thought to subserve semantic processing (i.e., left anterior temporal cortex). It could also be argued that, in the PD population, what has been labeled a negative priming effect may instead reflect underlying deficits in set-shifting (i.e., moving between the rule of “ignore” to “attend”), as these functions are known to be disrupted in this clinical population (for review see Kudlicka et al., 2011). Future investigations should consider administering comprehensive neurocognitive batteries that independently assess component abilities, in order to better understand the possible interaction between baseline executive function deficits and task execution.

The present study was unable to speak to the influence of dopaminergic medication on performance in the PD group. All but one of the PD participants were medicated when they completed the task. Previous studies of semantic processing have demonstrated differential performance in PD groups when on and off levodopa (Angwin et al., 2006, 2009; Pederzolli et al., 2008; Arnott et al., 2011). It is therefore possible that any deficit in inhibitory processing could have been ameliorated by medication. Furthermore, Pessiglione et al. (2005) have demonstrated that healthy individuals, as well as those with PD who *are* receiving dopamine replacement therapy, are able to temporally segregate mental deliberation from motor execution when making a decision between alternatives. This allows for the intervening influence of higher order cognitive processes such as memory and reasoning. However, in individuals with PD who are *not* receiving dopamine replacement therapy, mental deliberation and motor execution are coupled together in time, resulting in interference between the two processes. This interference manifests as slower movement time and increased evidence of movement hesitation. The relatively spared performance of the present PD group may therefore have been facilitated by their medicated state (though it should be noted that to date, this decoupling/coupling phenomenon has primarily been described in studies utilizing a manual response, rather than speech gestures). In addition, differences in the level of medication of PD participants (ranging from non-medicated to optimally medicated at time of testing) may explain the variable performance observed across different versions of the negative priming task, particularly for those studies that utilized manual, motor-based responses (e.g., the visuospatial/location priming administered by Stout et al., 2001; Filoteo et al., 2002; Wylie and Stout, 2002; Troche et al., 2006), and indeed across different tasks presumed to provide a measure of lexical-semantic inhibition. Future studies should endeavor to administer the present paradigm in individuals during both medicated and un-medicated states, in order to better understand the influence of disease pathology upon semantic inhibition. Amendments to the paradigm itself allowing for isolated observation of mental deliberation vs. motor response execution would also better inform conclusions regarding the locus of any resulting differences in performance.

A similar limitation in the present study concerns the progression of cognitive decline relative to stage of disease. The PD participants in the present study were all judged to be mildly-moderately affected by the disease. It is possible that cognitive processes such as the inhibition assessed here are relatively intact at this stage of the disease, as striatal dopamine depletion has yet to progress to those regions thought to be associated with these cognitive functions (Cools et al., 2001; Cools, 2006). Finally, the authors acknowledge that the self-paced approach utilized in the present task may have resulted in individual variations in SOA that could have influenced performance, hence future studies may benefit from stricter control of this variable.

Some evidence for the notion that individual mechanisms of attention/inhibition may exist for different cognitive domains has been generated by the results of the present study. However, obtaining conclusive support for this hypothesis will require systematic evaluation of each domain in isolation. Furthermore, it appears that within each domain, input modality and response requirements must be strictly controlled in order to tease apart the precise conditions under which different types of inhibition are evoked. In conclusion, the present study suggests that PD participants are largely unimpaired in their ability to suppress irrelevant semantic information evoked by a picture, and that this suppression is maintained across a one-trial interval. It can be further speculated that inhibition processes are subserved by specialized mechanisms unique to individual domains (e.g., visuospatial vs. lexical-semantic vs. visual-semantic), and these mechanisms may be differentially affected by the pathology of PD. These results suggest that inhibitory mechanisms related to the processing of visual-semantic stimuli may be largely intact in PD. Further investigation using paradigms that strictly control for the influence of lexical-semantic input and output is required in order to elucidate the integrity of such mechanisms.

## Data Availability

The raw data supporting the conclusions of this manuscript will be made available by the authors, without undue reservation, to any qualified researcher.

## Author Contributions

MI, KM, AA and DC contributed to the conception and design of the study. KM and MI developed computer-based experimental paradigms. MI acquired the baseline neurocognitive and experimental data. MI and DC performed the statistical analysis. MI wrote the first draft of the manuscript. All authors contributed to manuscript revision, read and approved the submitted version.

## Conflict of Interest Statement

The authors declare that the research was conducted in the absence of any commercial or financial relationships that could be construed as a potential conflict of interest.
